# Combined forced oscillation and forced expiration measurements in mice for the assessment of airway hyperresponsiveness

**DOI:** 10.1186/1465-9921-11-82

**Published:** 2010-06-21

**Authors:** Karim H Shalaby, Leslie G Gold, Thomas F Schuessler, James G Martin, Annette Robichaud

**Affiliations:** 1 Meakins Christie Laboratories, McGill University, Montreal (Qc), Canada; 2 SCIREQ Scientific Respiratory Equipment Inc., Montreal (Qc), Canada

## Abstract

**Background:**

Pulmonary function has been reported in mice using negative pressure-driven forced expiratory manoeuvres (NPFE) and the forced oscillation technique (FOT). However, both techniques have always been studied using separate cohorts of animals or systems. The objective of this study was to obtain NPFE and FOT measurements at baseline and following bronchoconstriction from a single cohort of mice using a combined system in order to assess both techniques through a refined approach.

**Methods:**

Groups of allergen- or sham-challenged ovalbumin-sensitized mice that were either vehicle (saline) or drug (dexamethasone 1 mg/kg ip)-treated were studied. Surgically prepared animals were connected to an extended *flexiVent *system (SCIREQ Inc., Montreal, Canada) permitting NPFE and FOT measurements. Lung function was assessed concomitantly by both techniques at baseline and following doubling concentrations of aerosolized methacholine (MCh; 31.25 - 250 mg/ml). The effect of the NPFE manoeuvre on respiratory mechanics was also studied.

**Results:**

The expected exaggerated MCh airway response of allergic mice and its inhibition by dexamethasone were detected by both techniques. We observed significant changes in FOT parameters at either the highest (Ers, H) or the two highest (Rrs, R_N_, G) MCh concentrations. The flow-volume (F-V) curves obtained following NPFE manoeuvres demonstrated similar MCh concentration-dependent changes. A dexamethasone-sensitive decrease in the area under the flow-volume curve at the highest MCh concentration was observed in the allergic mice. Two of the four NPFE parameters calculated from the F-V curves, FEV_0.1 _and FEF50, also captured the expected changes but only at the highest MCh concentration. Normalization to baseline improved the sensitivity of NPFE parameters at detecting the exaggerated MCh airway response of allergic mice but had minimal impact on FOT responses. Finally, the combination with FOT allowed us to demonstrate that NPFE induced persistent airway closure that was reversible by deep lung inflation.

**Conclusions:**

We conclude that FOT and NPFE can be concurrently assessed in the same cohort of animals to determine airway mechanics and expiratory flow limitation during methacholine responses, and that the combination of the two techniques offers a refined control and an improved reproducibility of the NPFE.

## Background

An excessive airway response to agonists such as methacholine (MCh) or histamine is widely employed as a diagnostic criterion for asthma [1]. Response is generally measured in human subjects through the spirometric assessment of maximal forced expiratory manoeuvres following the administration of progressively increasing concentrations of the constrictive agonist [1]. Forced expiratory manoeuvres have been favoured because of their relative technical simplicity and the widespread availability of inexpensive equipment. However, forced expirations are dependent on patient cooperation, which is not possible to obtain in very young patients [2], and techniques such as forced oscillatory mechanics [3] and the squeeze technique for forced expirations have been applied in these circumstances [4-6].

In experimental animals, airway responsiveness is commonly assessed using measurements of lung mechanics acquired during tidal breathing or using forced oscillation with volumes less than tidal volume. Forced expiratory manoeuvres have also been used to successfully assess airway hyperresponsiveness in the mouse [7-11] and rat [10,12]. In these experiments, rapid forced expiration was induced by subjecting the tracheostomized animals to a large negative pressure. Direct comparisons of the two approaches of measuring airway responsiveness have been reported in mice using either separate groups of animals or separate equipment. The objective of this study was to obtain lung function measurements at baseline and following bronchoconstriction from both techniques using a single cohort of mice and a single system. More specifically, negative pressure-driven forced expiratory (NPFE) and forced oscillation technique (FOT) manoeuvres were concurrently performed using a single combined setup in groups of allergen- or sham-challenged ovalbumin-sensitized mice. We studied the performance of these tests at baseline and following increasing aerosolized MCh challenges as well as in the context of a therapeutic intervention with dexamethasone, a drug known to inhibit allergen-induced airway hyperresponsiveness. The impact of NPFE on respiratory mechanics was investigated as well.

## Methods

### Animals

Six to eight week-old, female Balb/c mice, ranging in weight between 17 and 22 grams at the time of study, were purchased from Charles River, Canada. The mice were housed in a conventional animal facility under a 12 hour light/dark cycle with free access to food and water. Experimental procedures were approved by McGill University Institutional Animal Care Committee.

### Experimental procedures and protocol

Animals were divided in four experimental groups: (i) vehicle-treated, saline-challenged (Veh/Sal), (ii) dexamethasone-treated, saline-challenged (Dex/Sal), (iii) vehicle-treated, OVA-challenged (Veh/OVA), and (iv) dexamethasone-treated, OVA-challenged mice (Dex/OVA). All mice received two intraperitoneal (ip) sensitizations, one week apart (Day 0 and 7), of 10 μg ovalbumin (OVA grade V; Sigma-Aldrich, USA) and 1 mg aluminum hydroxide (Sigma-Aldrich, USA) in 0.2 ml sterile saline. The mice were challenged one week later on three consecutive days (Day 14, 15, 16) by intranasal instillation of either sterile saline, or 10 μg OVA/day (in 36 μl) under light isoflurane anesthesia. One day prior to OVA- or saline-challenge (Day 13), animals began receiving daily ip injections of either sterile saline (vehicle) or 1 mg/kg dexamethasone, until one day after the final challenge (Day 17). All measurements were obtained 48 hours following the final challenge (Day 18). On the day of the experiment, mice were weighed and anesthetized with an injection of xylazine hydrochloride (10 mg/kg, ip) followed 5 minutes later by the administration of sodium pentobarbital (32 mg/kg, ip). Once the desired level of anesthesia was reached, as assessed by loss of withdrawal reflex and absence of response to external stimuli, the mouse was tracheostomized using an 18G metal cannula. The animal was then placed in a flow-type body plethysmograph and connected via the endotracheal cannula to a *flexiVent *system (SCIREQ Inc., Montreal, Canada). After initiating mechanical ventilation, the mouse was paralyzed with a 1 mg/kg pancuronium bromide ip injection and subjected to a deep lung inflation (DI; slow inflation to a pressure of 30 cmH_2_O held for 3 seconds) before the plethysmograph was sealed for the rest of the experiment. The animal was ventilated at a respiratory rate of 150 breaths/min and tidal volume of 10 ml/kg against a positive end expiratory pressure (PEEP) of 3 cmH_2_O.

### Experimental Setup

**Figure 1:**
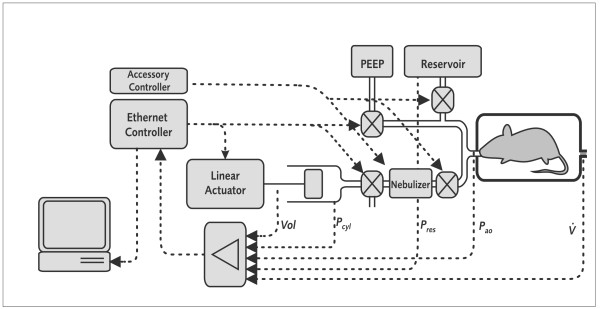
**Block diagram of *flexiVent *system with extensions for negative pressure-driven forced expiration manoeuvres**. During a negative pressure-driven forced expiration manoeuvre, the reservoir pressure (P_res_) as well as the air flow into the plethysmograph () were recorded via precision differential pressure transducers attached respectively to the negative pressure reservoir and the pneumotachograph mounted on the plethysmograph chamber. These signals were collected in addition to the volume displaced by the piston (Vol), the pressure in the cylinder (P_cyl_) and the pressure at airway opening (P_ao_) typically recorded by the *flexiVent*. PEEP stands for positive end expiratory pressure.

To permit NPFE and FOT measurements in the same setup, we extended a standard *flexiVent *system as follows (Figure 1). The inspiratory arm of the Y-tubing contained a computer-controlled nebulizer (Aeroneb Lab, standard mist model, Aerogen Ltd, Ireland) as well as a computer-operated pinch valve that isolated the nebulizer from high negative pressures during NPFE manoeuvres. A T-piece in the expiratory limb of the ventilator connected the mouse airways to a negative pressure reservoir via a second computer-operated fast response (typical opening time < 4 ms) solenoid shutter valve. The reservoir pressure and the air flow into the plethysmograph were recorded during NPFE manoeuvres via precision differential pressure transducers attached, respectively, to the pressure reservoir (SCIREQ UT-PDP-100; 10 kPa nominal range) and the pneumotachograph mounted on the plethysmograph chamber (SCIREQ UT-PDP-02; 0.2 kPa nominal range). This was done in addition to the signals typically recorded by the *flexiVent*, i.e. volume displaced by piston, pressure in the cylinder and pressure at airway opening. All data were digitized at a rate of 256 Hz with 12 bit accuracy. The mechanical cut-off frequency of the plethysmograph chamber was over 300 Hz. The spectra of the forced expired flow signals we collected did not contain any significant power at frequencies above 50 Hz.

### Forced Oscillation Measurements

Respiratory mechanics were assessed using a 1.2 second, 2.5 Hz single-frequency forced oscillation manoeuvre (SFOT; using the SnapShot-150 perturbation) and a 3 second, broadband low frequency forced oscillation manoeuvre containing 13 mutually prime frequencies between 1 and 20.5 Hz (LFOT; using the Quick Prime-3 perturbation). The settings of both perturbations were configured to ensure that onset transients were omitted and the oscillations had reached steady state in the analyzed portions of the manoeuvres. Respiratory system resistance (Rrs) and elastance (Ers) were calculated in the *flexiVent *software by fitting the equation of motion of the linear single compartment model of lung mechanics to the SFOT data using multiple linear regressions. Respiratory system input impedance was calculated from the LFOT data and Newtonian resistance (R_N_), tissue damping (G) and tissue elastance (H) were determined by iteratively fitting the constant-phase model [13] to input impedance. Both FOT manoeuvres were executed every 15s in alternation after each MCh aerosol challenge to capture the time course and the detailed response of the MCh-induced bronchoconstriction.

### Forced Expiratory Measurements

In preparation for each NPFE manoeuvre, the negative pressure reservoir was adjusted to a given negative target pressure by retracting a sufficiently large syringe. Once the manoeuvre was initiated, the *flexiVent *was programmed to gradually inflate the mouse lungs to a pressure of 30 cmH_2_O over 1 second and hold this pressure for 2 seconds before opening the shutter valve to connect the animal's airway opening to the negative pressure reservoir for 2 seconds. The negative pressure gradient generated a rapid deflation of the mouse lungs and the ensuing flow of air into the body box associated with the animal chest wall movement was measured. From that signal, we calculated the forced expired volume over 0.1 second (FEV_0.1_), forced vital capacity (FVC), peak expiratory flow (PEF) and forced expiratory flow at 50% of FVC (FEF50). In order to study the relationship between the expiratory flow and the driving pressure, we repeated this procedure with increasingly negative pressures in 10 cmH_2_O increments from -15 to -65 cmH_2_O.

### Impact of NPFE on respiratory mechanics

During pilot experiments and to assess the effect of NPFE manoeuvres on lung function, we measured respiratory mechanics using the FOT immediately and 1, 3, 5 and 10 minutes after a NPFE manoeuvre performed with a reservoir pressure of -35 cmH_2_O and a PEEP of 2 cmH_2_O. Then, we administered a DI and obtained another set of FOT data. Similar data were obtained in our main experiments in OVA-sensitized, vehicle pre-treated, sham-or OVA-challenged animals over a time frame of one minute after NPFE performed with a reservoir pressure of -55 cmH_2_O and a PEEP of 3 cmH_2_O.

### Assessment of allergen-induced airway hyperresponsiveness by FOT and NPFE

**Figure 2:**
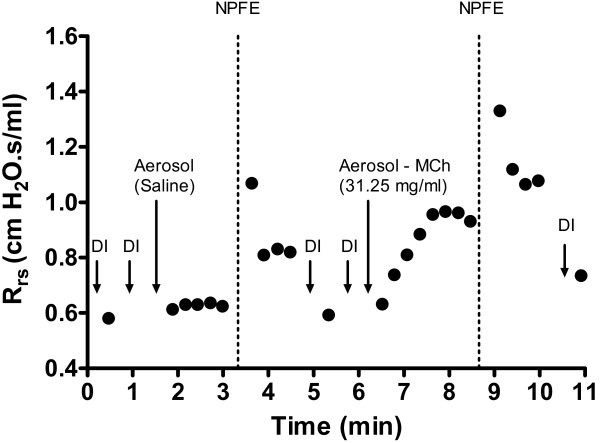
**Measurement protocol**. Experimental trace in a sham control mouse illustrating the timing of a negative pressure-driven forced expiration (NPFE) manoeuvre following saline and methacholine (31.25 mg/ml) aerosol challenge, using closely-spaced (15s) single-frequency forced oscillation parameter Rrs to follow the time-course of the response. Rrs = respiratory system resistance; DI = deep lung inflation (30 cmH_2_O); MCh = methacholine.

Following DI and baseline measurements, saline solution was delivered to the mouse as an aerosol using a 4s nebulization period synchronized with inspiration at a nebulization rate of 50%. FOT measurements were then used to monitor the time-course of the ensuing response, as described above. Immediately upon observing a peak in Rrs reported by the software, a single NPFE manoeuvre was applied, as previously described, using a negative pressure of -55 cmH_2_O. Measurements of FOT parameters resumed immediately following the NPFE manoeuvre for a period of one minute. To ensure a return to baseline, the mouse underwent repeated DIs followed by default ventilation and respiratory mechanics measurements prior to the administration of an initial MCh-induced bronchoprovocation (31.25 mg/ml acetyl-β-methylcholine; Sigma-Aldrich, USA). In this manner, doubling concentrations of MCh were administered up to 250 mg/ml and a NPFE manoeuvre was performed at the peak response to a given concentration (Figure 2).

### Statistical analysis

The results are expressed as mean ± SD with *n *being the number of animals per group. For statistical analyses, responses were converted to their logarithms (log_10_) and differences between groups were analysed using analyses of variance for repeated measurements (ANOVA) followed by Bonferroni or Tukey's multiple comparisons with *p *< 0.05 considered statistically significant (GraphPad Prism version 5; GraphPad Software, San Diego, USA) [14].

## Results

### Impact of NPFE on respiratory mechanics

**Figure 3:**
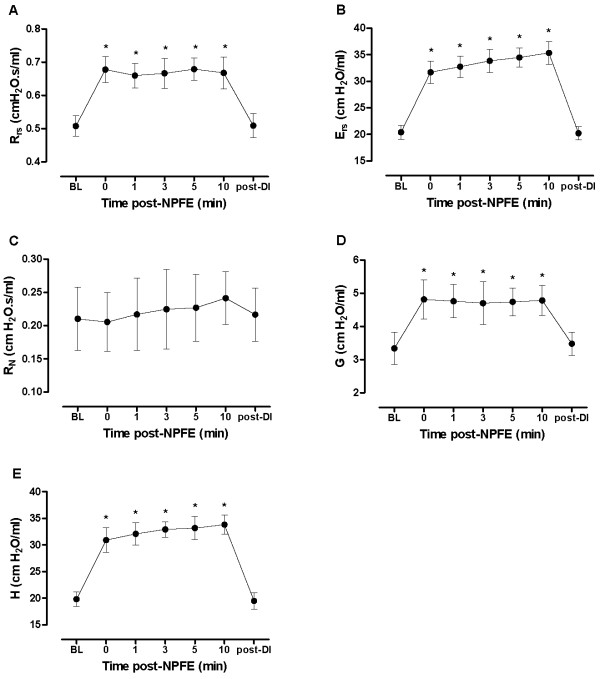
**Impact of negative pressure-driven forced expiration manoeuvres on respiratory mechanics**. Respiratory mechanics in naïve mice at baseline (BL), following the application of a negative pressure-driven forced expiration (NPFE) manoeuvre and following deep lung inflation (post-DI; 30 cmH_2_O). Values are mean ± standard deviation from a group of 12 mice that were each studied once in the absence of methacholine challenge. (*p < 0.05; ANOVA).

Following the application of a negative pressure to perform NPFE manoeuvres in naïve mice in the absence of MCh challenge (-35 cmH_2_O and a PEEP of 2 cmH_2_O), we observed a sustained increase in Rrs, Ers, G and H, but not in R_N _(Figure 3). This effect did not spontaneously reverse during a period of 10 minutes of tidal ventilation, but respiratory mechanics returned to baseline after DI. Given this impact of NPFE manoeuvres on lung mechanics in our pilot experiments, DI was performed following all subsequent NPFE manoeuvres. We also investigated whether the effect of the NPFE manoeuvre on respiratory mechanics was amplified in vehicle-treated allergen-sensitized and challenged animals studied for one minute post-NPFE manoeuvre following saline aerosol administration. The adverse effect of the NPFE was reproduced, but not significantly augmented, in OVA-challenged, compared to sham-challenged mice.

### The pressure-dependence of expiratory flow

**Figure 4:**
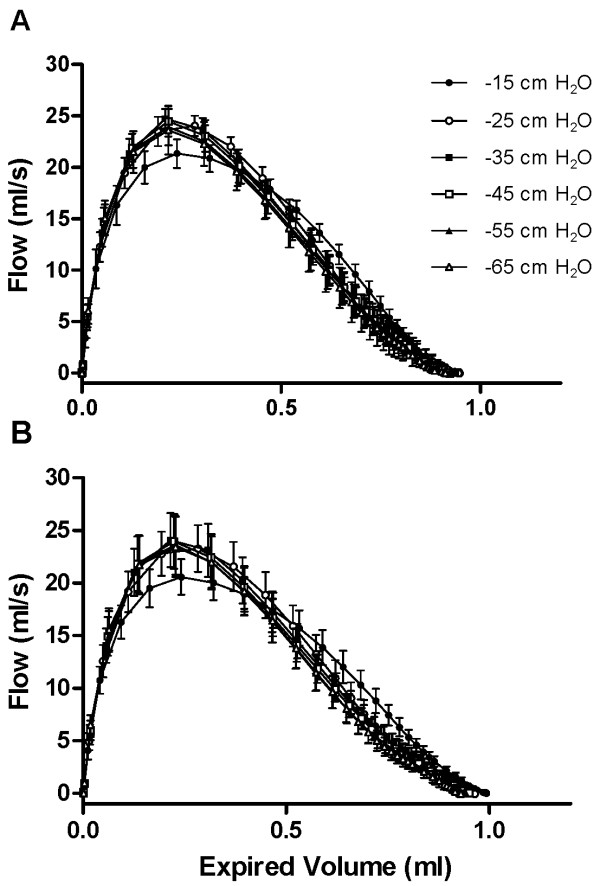
**Pressure-dependence of expiratory flow**. Mean flow-volume curves from ovalbumin-sensitized and sham-challenged mice (A) and ovalbumin-sensitized and challenged mice (B) at varying negative pressures. Values are mean ± standard deviation from groups of 7-9 mice (1 determination per animal at each negative pressure).

Mean flow-volume curves obtained over a range of negative pressures from -15 to -65 cmH_2_O, for both sham-challenged and OVA-challenged allergic mice are shown in Figure 4. As expected, the mean peak expiratory flow was pressure-dependent at lower pressures. Negative pressures of -15 and, to a lesser extent, -25 cmH_2_O produced sub-maximal peak expiratory flows and altered flow-volume loops when compared to higher pressures both in sham- and OVA-challenged mice. In sham-challenged, as well as, in OVA-challenged mice, negative pressures of -35, -45, -55 and -65 cmH_2_O produced virtually identical flow-volume loops, indicating that maximal expiratory flow had been reached. In all subsequent NPFE manoeuvres, a negative pressure of -55 cmH_2_O was used to ensure that a maximal effect was evoked.

### Assessment of airway responsiveness to methacholine

**Figure 5:**
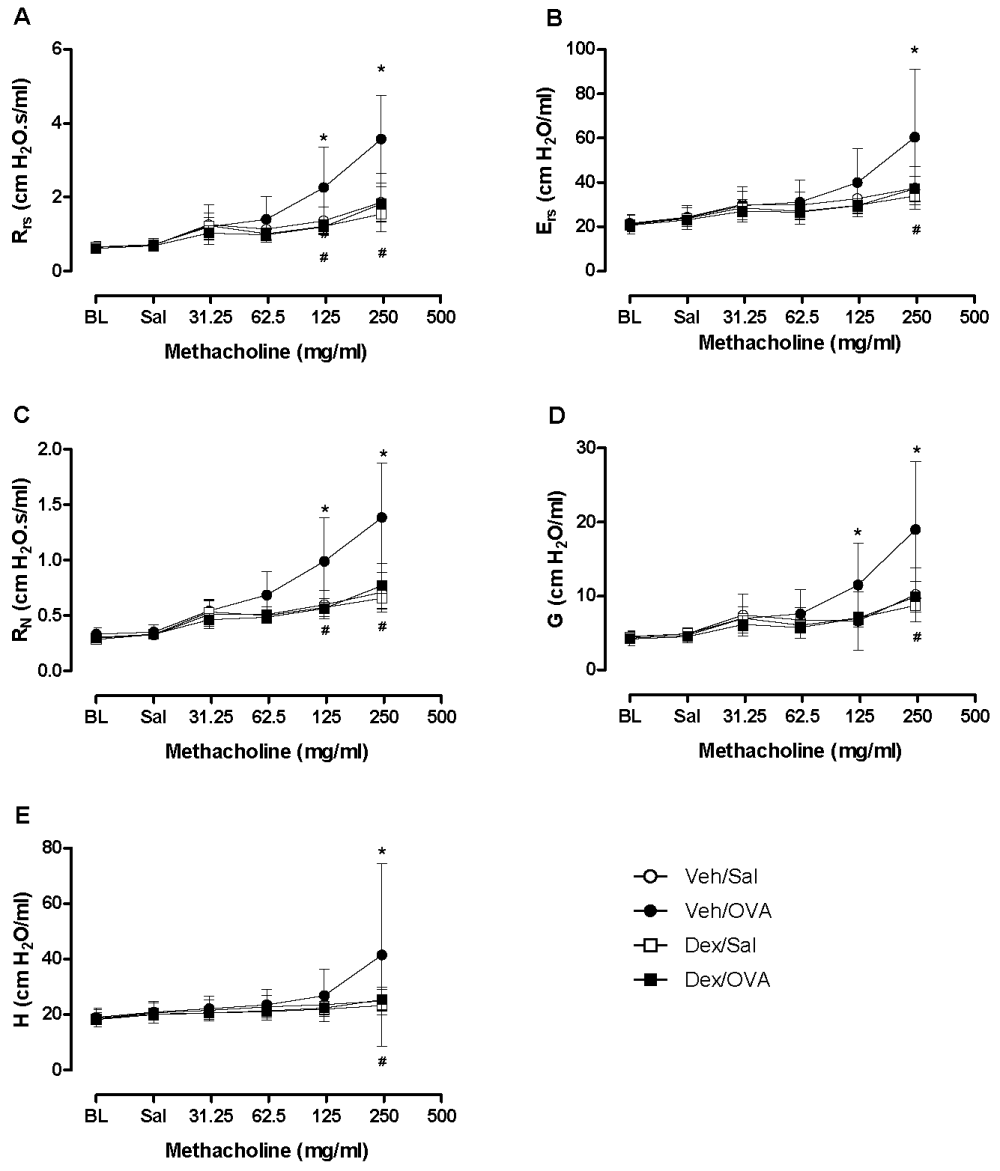
**Assessment of allergen-induced airway hyperresponsiveness by the forced oscillation technique**. Forced oscillation parameters at peak Rrs response to each concentration of aerosolized methacholine in ovalbumin- and saline-challenged OVA-sensitized mice that were either vehicle- or dexamethasone-treated. Values are mean ± standard deviation from groups of 5-7 mice. (*p < 0.05 Veh/OVA vs Veh/Sal, ^#^*p *< 0.05 Veh/OVA vs Dex/OVA; ANOVA).

**Figure 6:**
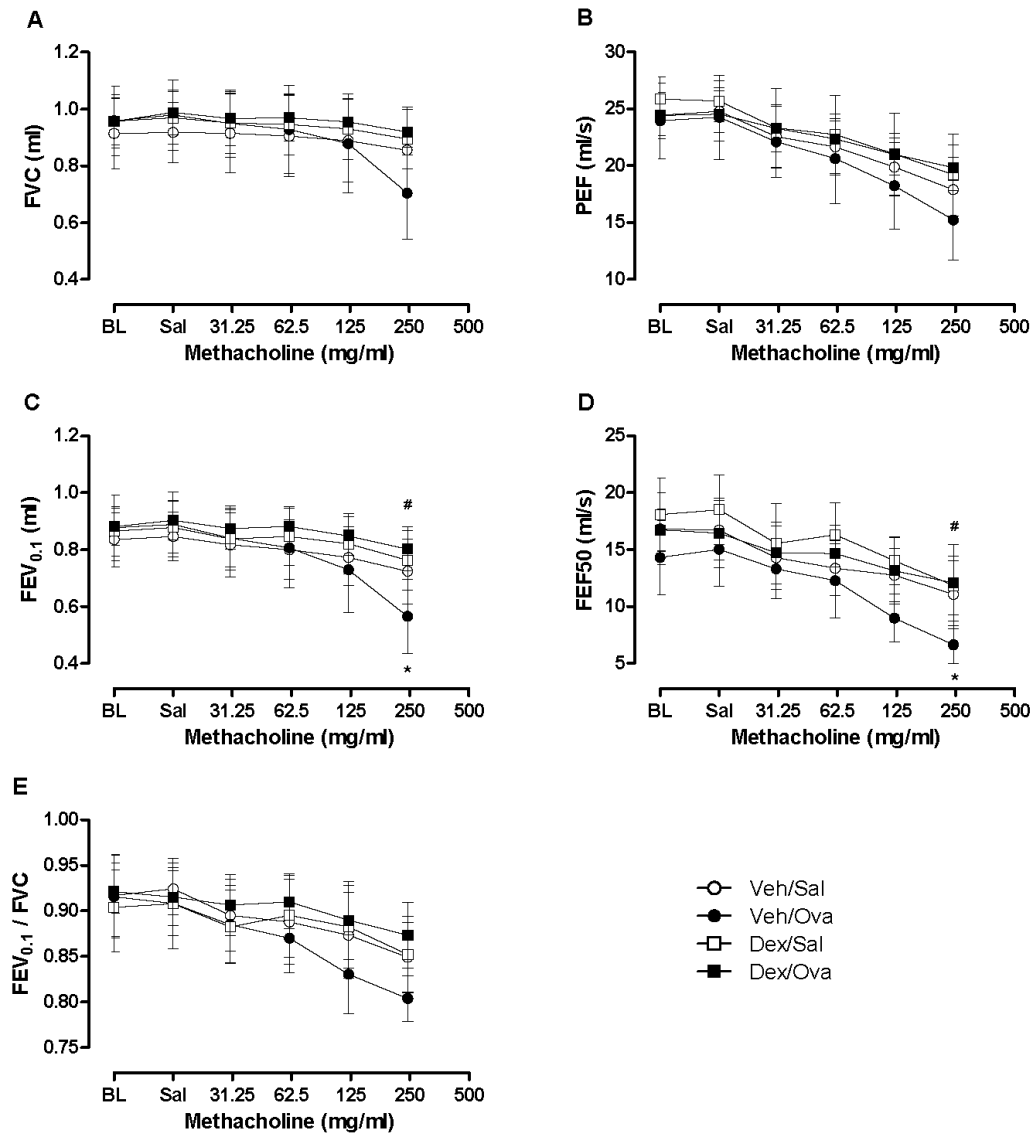
**Assessment of allergen-induced airway hyperresponsiveness by negative pressure-driven forced expiratory parameters**. Forced expiration parameters at baseline (BL) and following aerosolized saline (Sal) or increasing methacholine concentrations in vehicle (Veh)- or dexamethasone (Dex)-treated, sham (Sal)- and ovalbumin (OVA)-challenged ovalbumin-sensitized mice. Values were obtained at peak response to each concentration of aerosolized methacholine and are expressed as mean ± standard deviation from groups of 4-6 mice where each animal was studied once. (*p < 0.05 Veh/OVA vs Veh/Sal, ^#^*p *< 0.05 Veh/OVA vs Dex/OVA; ANOVA).

Mean baseline lung function parameters for the different experimental groups did not differ significantly whether assessed by FOT or by NPFE parameters (Figures 5 and 6). However, as expected, the group of OVA-challenged allergic mice demonstrated a dexamethasone-sensitive hyperresponsiveness to MCh compared to its respective control group, as illustrated by significant increases in all FOT parameters after the 125 and/or 250 mg/ml aerosol bronchoprovocation and reversal following drug treatment (Figure 5).

**Figure 7:**
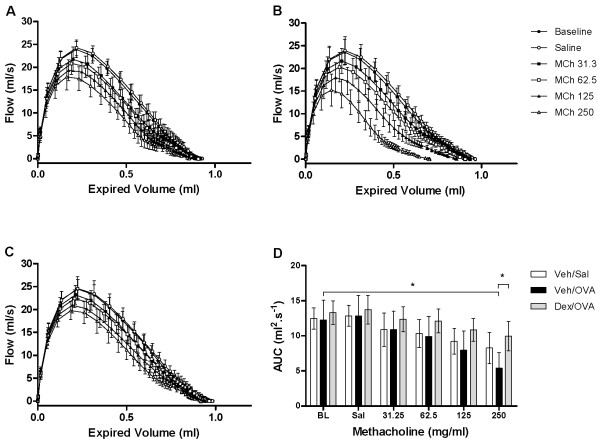
**Flow-volume curves following increasing aerosolized methacholine concentrations**. Mean flow-volume curves (mean ± standard deviation) from vehicle-treated saline- (A; Veh/Sal) and ovalbumin- (B; Veh/OVA) challenged ovalbumin-sensitized mice as well as from dexamethasone (1 mg/kg)-treated ovalbumin-sensitized and challenged mice (C; Dex/OVA) at baseline (BL) and following aerosolized saline (Sal) or increasing methacholine concentrations (MCh 31.25-250 mg/ml). Figure 7D represents the mean and standard deviation of the area under the flow-volume curves (AUC) under the varied experimental conditions. (*p < 0.05; ANOVA, *n *= 5-6 mice/group).

**Figure 8:**
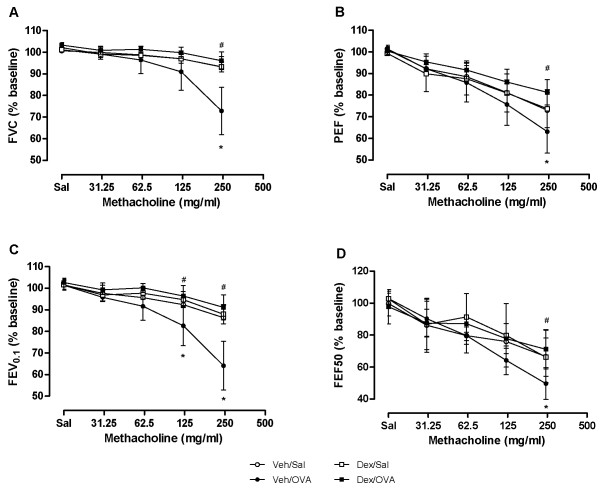
**Normalized forced expiratory parameters**. Forced expiration parameters normalized to baseline values at each concentration of aerosolized methacholine in ovalbumin-challenged (OVA) and sham-challenged (Sal) ovalbumin-sensitized mice that were either vehicle (Veh)- or dexamethasone (Dex)-treated. Values were normalized to individual baseline and expressed as mean ± standard deviation for each group (*n *= 4-6 mice/group, each mouse studied once). (*p < 0.05 Veh/OVA vs Veh/Sal; ^#^*p *< 0.05 Veh/OVA vs Dex/OVA; ANOVA).

**Figure 9:**
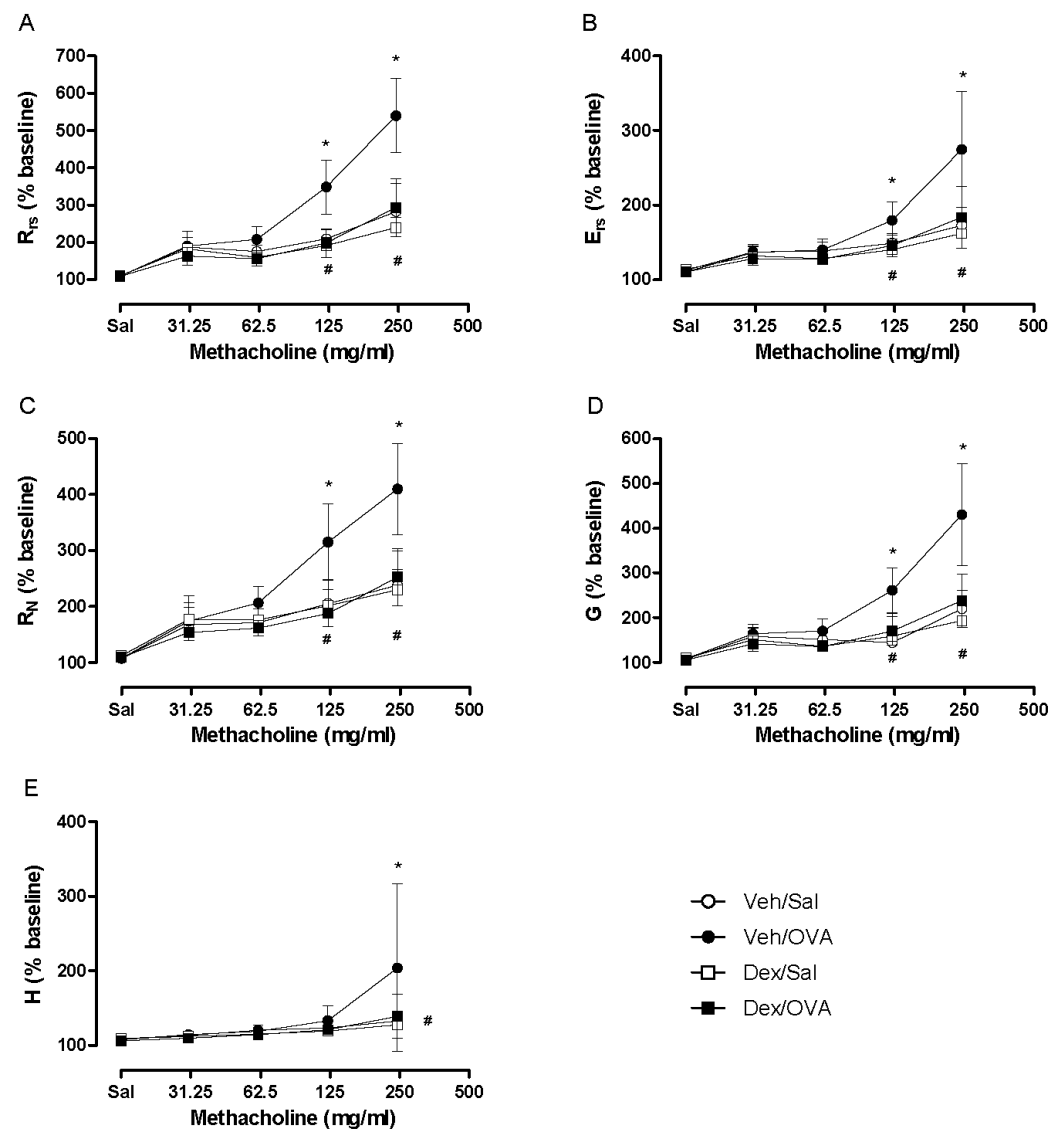
**Assessment of drug effect via normalized parameters of the forced oscillation technique**. Forced oscillation technique parameters normalized to baseline values at each concentration of aerosolized methacholine in vehicle treated- (Veh/OVA; closed circles) and dexamethasone treated- (Dex/OVA; closed squares) ovalbumin sensitized and challenged mice. Values were normalized to individual baseline and expressed as mean ± standard deviation. (*p < 0.05 Veh/OVA vs Veh/Sal; ^#^*p *< 0.05 Veh/OVA vs Dex/OVA; ANOVA; *n *= 5-7 mice/group).

The flow-volume curves obtained from NPFE manoeuvres also demonstrated MCh concentration-dependent changes with a decrease in the area under the flow-volume curve that was more pronounced at the highest MCh concentration in the OVA-challenged allergic mice and reversible by dexamethasone treatment (Figure 7D). From the four NPFE parameters calculated, an exaggerated response to methacholine was significantly detected in the OVA-challenged mice with FEV_0.1 _and FEF50 at the highest concentration (Figure 6C, D). Normalization of FEV_0.1 _to FVC extracted from the same manoeuvre did not improve the sensitivity with which airway hyperresponsiveness was detected (Figure 6E). However, normalization to baseline permitted hyperresponsiveness of the OVA-challenged mice relative to the sham-challenged animals (Veh/OVA vs Veh/Sal) to be detected at a lower concentration of MCh (125 mg/ml) (Figure 8C). Also, when NPFE parameters were expressed as % of baseline, airway hyperresponsiveness of the OVA-challenged mice was captured by all four parameters calculated but mostly at the highest MCh concentration (Figure 8). Normalization to baseline had a minimal impact on FOT results (Figure 9). Finally, the effect of the drug treatment on preventing airway hyperresponsiveness (Dex/OVA vs Veh/OVA) was detected by both techniques (Figures 5, 6, 7, 8, and 9).

## Discussion

In this study, we obtained measurements of NPFE and FOT from the same cohorts of animals using a setup that combined both techniques. NPFE manoeuvres in mice, unlike spirometry in humans, are invasive procedures. As with FOT measurements, NPFE manoeuvres require that the animals undergo anaesthesia, tracheotomy or intubation, and mechanical ventilation. The combination of the two techniques in a single set-up allowed us to study the performance of both tests through a refined approach. In the present study, we measured airway responsiveness to MCh in a mouse model of allergen-induced airway hyperresponsiveness using concurrent NPFE and FOT manoeuvres and examined whether one technique offered practical advantages or was informative in ways that the other was not.

As expected, we found forced expiration to be pressure-dependent at lower negative pressures but pressure-independent at higher negative pressures. In our animals, a negative pressure of -35 cmH_2_O or greater was required to reliably produce a maximal forced expiration (Figure 4). Above this threshold, expiratory flow became independent of the driving pressure, indicating that maximal flow was produced and that expiratory flow limitation (EFL) played an important role in determining the forced expiratory flow.

In the present model of allergen-induced airway hyperresponsiveness, the four experimental groups studied were indistinguishable under baseline conditions by FOT or NPFE. Baseline values of calculated parameters from either measurement technique were comparable to those reported in the literature (Figures 5, 6) [7,9,11].

Under our experimental conditions, we were able to detect airway hyperresponsiveness to MCh in vehicle-treated allergen-challenged mice compared to the sham-challenged or drug-treated mice by both techniques. In addition to significant increases in FOT parameters following MCh provocation, we also observed significant changes when using the NPFE technique. Therefore, we found, as in previous studies [7-12], that NPFE can be used as an indicator of bronchoconstriction in mice.

However, compared to FOT, the sensitivity at which NPFE parameters significantly detected the MCh-induced changes was lower. Normalization to baseline improved this sensitivity while having minimal impact on FOT responses. This discrepancy could highlight the fact that the two measurement techniques are determined by different factors or alternatively, that the distribution of a specific determinant of NPFE (perhaps lung volumes) was unequal between groups and that the normalization of NPFE parameters in terms of the initial lung condition provided an adjustment [15]. Since good statistical practice in pharmacology generally recommends looking at data in its raw form before any normalization [16], our results highlight a potential shortcoming of the NPFE technique compared to FOT. Normalization to baseline could prove to be difficult in chronic or longitudinal studies where baseline recordings are collected an extended period of time before the measurements.

The interpretation and structural correlation of human spirometry is fairly complex since it has been shown to be influenced by a variety of factors, including upper airway resistance, EFL, elastic lung and chest wall recoil, patient characteristics, health status or effort [17]. However, not all these confounding factors apply to the NPFE manoeuvres we performed in mice since some were controlled by the machine or the experimental protocol. The animals were anaesthetized, tracheostomized and passive, so their upper airways were bypassed and effort or muscular pressure was eliminated. Furthermore, prior to a forced expiration manoeuvre, the mouse lungs were inflated to a controlled and highly reproducible inflation pressure of 30 cmH_2_O, which contributed to standardize the driving pressure for the manoeuvre, to minimize the variations in elastic recoil and to achieve maximal expiration. This leaves EFL as one of the remaining factors governing the flow-volume loops obtained from NPFE in mice. While DI contributes in this manner to lower variability between animals, it may influence the magnitude of the MCh-induced bronchoconstriction by opening airways immediately prior to the forced expiration, which would be expected to reduce the airway resistance [18].

In previous assessments of airway responsiveness by NPFE, manoeuvres were often performed at pre-determined times following MCh administrations [8,9,11]. In the present study, the combination with FOT allowed us to measure respiratory mechanics in real-time leading up to, and following the NPFE manoeuvre, thus avoiding added variance related to the timing of the NPFE measurement. Consequently, reproducible flow-volume curves with relatively small within-group variability were obtained, compared to what has been previously reported [7,11], despite the small group sizes and single NPFE manoeuvres that were used.

Using closely spaced (15 seconds apart) repeated FOT measurements to capture the physiological response to the inhaled MCh challenge, Rrs was used to select the moment at which the NPFE manoeuvre was performed. However, in addition to the ability to follow the time-course of the bronchoconstrictor response, FOT also offers the possibility to distinguish between central and peripheral respiratory mechanics. The mathematical models used in the analysis of FOT data, specifically the constant-phase model [13], can provide valuable information pertaining to the heterogeneity of the respiratory response and whether it is dominated by resistance of the conducting airways, peripheral airway closure or tissue resistance [19]. Ultimately, any FOT parameter could serve as a guide to refine the experimental design.

The combination of both techniques in a single setup also allowed us to study the impact of NPFE on respiratory mechanics and to investigate the underlying mechanisms. Our data indicated that the NPFE manoeuvre itself affected the respiratory mechanics (Figure 3). Namely, it caused a significant increase in all FOT parameters, except R_N_. The proportional increases in G and H suggest that the NPFE manoeuvre causes a uniform derecruitment of peripheral lung units [20]. R_N _represents the resistance of the conducting airways, which is dominated by the larger proximal airways. Therefore, this finding suggests that the loss of lung units is restricted to the periphery, possibly caused by small airway closure or alveolar collapse. It is interesting to note that while R_N _was not altered following a NPFE manoeuvre, Rrs was. Since Rrs is still commonly interpreted as a surrogate of airway resistance, it is worth pointing out that our finding that Rrs is altered under these circumstances confirms that this parameter is also coupled to the resistive properties of the lung tissues and that therefore it does not solely reflect airway resistance.

The sustained airway closure caused by NPFE was reversible by deep inflation. Thus, for the assessment of MCh responsiveness, this limitation of the technique was addressed by performing DI following each NPFE manoeuvre to ensure automated and reproducible lung recruitment. However, the post-NPFE DI interfered with the ability to perform closely spaced repeated NPFE measurements, to measure cumulative bronchoconstrictor dose-responses to MCh using NPFE or to follow by FOT the course of the bronchoconstrictive response after a NPFE manoeuvre.

Expiratory flow limitation is a major nonlinear effect in the lungs that may play an important role in many disease models. In the current study, the NPFE data mostly mirrored the FOT data and provided no complementary information. However, Vanoirbeek *et al*. [11] recently reported EFL at baseline in an emphysematous mouse model, indicating that EFL assessment may prove valuable for other protocols and disease models. It is worth mentioning that the pattern of methacholine responsiveness observed in the present study differed from previous reports where a dominant peripheral lung response was noted following challenge in a similar model of allergen-induced airway hyperresponsiveness [14,19,21]. While previous studies also employed the forced oscillation technique to assess airway responsiveness, different nebulizers and nebulisation patterns were used in addition to variations in the ventilation circuitry around the nebulizer. These variations may account for the discrepancies as the intra-pulmonary dose of methacholine and/or its site of deposition could have been influenced.

Finally, although the extracted NPFE parameters in mice resemble those obtained in humans, the abovementioned differences in how these measurements are obtained in both species are sufficiently important that caution should be applied when directly comparing their outcomes, limitations or shortcomings until the validity of such comparisons has been established.

## Conclusions

In summary, we obtained concurrent FOT and NPFE measurements from the same cohort of mice using an extended *flexiVent *system that combined both techniques with the aim of assessing allergen-induced airway hyperresponsiveness as well as post-NPFE respiratory mechanics. The allergen-induced changes in lung function and their prevention by dexamethasone were detected by parameters of both techniques. Although in the context of the current protocol, NPFE provided no complementary information over and above FOT, NPFE as a method to assess EFL ultimately may complement FOT. Studying the mechanisms of NPFE-induced changes in respiratory mechanics broadened our understanding of the manoeuvre and allowed us to improve the way measurements were performed in order to get meaningful results. The combination of the two techniques represents an experimental design refinement applicable to a variety of respiratory disease models.

## Abbreviations

ANOVA: analysis of variance; DI deep lung inflation; EFL: expiratory flow limitation; Ers: respiratory system elastance; FEF50: forced expiratory flow at 50% of forced vital capacity; FEV_0.1_: forced expired volume over 0.1 second; FVC: forced vital capacity; FOT: forced oscillation technique; G: tissue damping; H: tissue elastance; LFOT: broadband low frequency forced oscillation technique; ip: intraperitoneal; MCh: methacholine; NPFE: negative pressure-driven forced expiration; OVA: ovalbumin; PEEP: positive end expiratory pressure; R_N_: Newtonian resistance; Rrs: respiratory system resistance; s: second; SFOT: single-frequency forced oscillation technique.

## Competing interests

LGG, TFS and AR are employed by SCIREQ Scientific Respiratory Equipment Inc. TFS also owns stock. KHS and JGM declare that they have no competing interests.

## Authors' contributions

KHS participated in the design of the study, the data acquisition and interpretation, drafted the manuscript and revised it critically for scientific content. LGG participated in the conception of the extended *flexiVent *system functionalities, data acquisition, analysis and interpretation, and revised the manuscript critically for scientific content. TFS conceived the extended *flexiVent *system, designed part of the study, participated in the data acquisition, performed the NPFE signal analyses and participated in result interpretation, manuscript writing and critical revision. JGM participated in the study design, the interpretation of results, manuscript writing and critical revision. AR designed part of the study, participated in the data acquisition, analysis and interpretation, and took part in manuscript writing and critical revision. All authors read and approved the final manuscript.
